# Environmental DNA: Preliminary Characterization of Microbiota in Three Sicilian Lakes

**DOI:** 10.1002/ece3.71276

**Published:** 2025-04-24

**Authors:** Manuela Mauro, Valeria Villanova, Mario Lo Valvo, Rosa Alduina, Slobodanka Radovic, Aiti Vizzini, Grazia Orecchio, Francesco Longo, Rosario Badalamenti, Vincenzo Ferrantelli, Gaetano Cammilleri, Annamaria Mauro, Vincenzo Arizza, Mirella Vazzana

**Affiliations:** ^1^ Department of Biological, Chemical and Pharmaceutical Sciences and Technologies (STEBICEF) University of Palermo Palermo Italy; ^2^ NBFC, National Biodiversity Future Center Palermo Italy; ^3^ IGA Technology Services Srl Udine Italy; ^4^ Istituto Zooprofilattico Sperimentale della Sicilia “A. Mirri” Palermo Italy; ^5^ Arpa Sicilia UOC Acque Interne e Suolo Palermo Italy

**Keywords:** eDNA, freshwater ecosystems, metabarcoding analysis, microbiota

## Abstract

Freshwater ecosystems play a key role in biogeochemical cycles and face various anthropogenic impacts. Understanding microbial communities within these ecosystems is crucial for ecosystem management and conservation. This study conducted a metabarcoding analysis of the bacterial 16S rDNA of environmental DNA (eDNA) to investigate the microbial diversity and composition in three Sicilian lakes (Poma, Piana, and Scanzano). The results revealed a common core microbiota in all the lakes, with Poma and Piana showing similar compositions, followed by Scanzano. Key bacterial phyla identified as core members across the sampled sites included Actinobacteriota, Proteobacteria, Bacteroidota, Verrucomicrobiota, Planctomycetota, and Cyanobacteria. Additionally, specific bacterial families such as Comamonadaceae, Burkholderiaceae, Ilumatobacteraceae, and Sporichthyaceae uniquely contributed to the microbial community structures. eDNA demonstrates potential as a quicker and less invasive tool to investigate biodiversity studies for these sites. The findings illuminate the intricate microbial dynamics within Sicilian freshwater ecosystems and emphasize the importance of considering microbial diversity in freshwater conservation and management strategies.

## Graphical abstract

Microbial content depends on the origin of the sample. This figure shows the relative abundance of bacteria in three lakes (Poma, Piana, and Scanzano) in Sicily, giving the first insights into microbial communities residing in those environments.

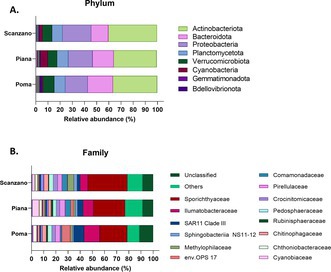

## 1 Introduction

In freshwater ecosystems, microorganisms play crucial functional roles, including regulating biogeochemical cycles and influencing the absorption and emission of greenhouse gases (Raymond et al. 2013; Weiman 2015; Crevecoeur et al. 2019; Meng et al. 2022; Ren et al. 2023). Climate change and anthropic activities are altering the biodiversity of these ecosystems, and for this reason, it is urgent to understand if these factors may impact microbiota and subsequently influence the biogeochemical cycles and ecosystem functions (Cavicchioli et al. 2019). For example, one of the well‐known phenomena is eutrophication, which is caused by an excess supply of nutrients from anthropic activities (e.g., urban development, agriculture, forestry and fishing industries) (Smith 1998; Carpenter et al. 1999). Although various physical and chemical techniques exist to determine the microbiota and the quality of water, they are often impractical because they are time‐consuming and expensive (Kenefick et al. 1993; He et al. 2016). In this context, the analysis of environmental DNA (eDNA) has emerged as a useful tool for the identification and monitoring of organisms, assessing biodiversity, and evaluating the health status of aquatic ecosystems (Deutschmann et al. 2019). eDNA is the genetic material present in the environment, originating from various sources such as skin, mucous membranes, saliva, sperm, secretions, eggs, feces, urine, blood, as well as from unicellular microorganisms and plant parts like leaves, fruits, or pollen (Ruppert et al. 2019; Taberlet et al. 2012; Bohmann et al. 2014).

Once extracted, eDNA undergoes high‐throughput next‐generation sequencing to detect short DNA fragments for species identification and differentiation of various taxa, a process commonly referred to as metabarcoding (Ruppert et al. 2019; Hebert and Gregory 2005; Hajibabaei et al. 2006). Among several conventional methodologies for the study of biotic communities, the analysis of eDNA holds significant importance (Deiner et al. 2016; Yang et al. 2017; Taberlet et al. 2018; Li et al. 2020, 2021; Altermatt et al. 2020). Within the realm of eDNA analysis, metabarcoding emerges as a powerful technique for unraveling the species diversity present in environmental samples, providing large‐scale spatial resolution and high‐quality levels of biomonitoring. The metabarcoding technique involves the extraction and analysis of genetic material from environmental samples such as water and soil that can target regions such as the 16S rDNA gene for bacterial identification (Bista et al. 2018; Ji et al. 2020). In this context, the approach of eDNA could be a reliable method for monitoring biodiversity, especially in freshwater systems, offering significant advantages over classical methods in terms of sampling invasiveness and time efficiency (Deiner et al. 2016; Taberlet et al. 2018; Thomsen and Willerslev 2015). Despite being introduced in the field of microbiology by Ogram et al. (1987), the eDNA technology remains widely utilized in various contexts, including the identification of sensitive species or pathogens (Coble et al. 2019; Robson et al. 2016). This approach allows researchers to study and identify bacterial diversity in ecosystems, providing insights into microbial community composition and dynamics and giving access also to the genetic information of uncultivable microorganisms. Despite the Mediterranean region being renowned as a biodiversity hotspot with diverse freshwater ecosystems and high levels of endemic and rare taxa, especially on Mediterranean islands like Sicily (de Tierno Figueroa et al. 2013), there has been limited focus on eDNA analysis of freshwater ecosystems in the region, with existing studies primarily concentrating on invertebrate and vertebrate taxa (Hupało et al. 2022; Mauro et al. 2023).

Here, we aimed to evaluate the microbial communities associated with different freshwater environments. Specifically, three artificial Sicilian lakes: Poma, Piana Albanesi, and Scanzano. The lakes were chosen as part of a research project that aims to characterize the biodiversity of the lakes in the province of Palermo (Sicily) through the use of eDNA analysis. The results of this study could significantly advance our understanding of the microbiota in freshwater ecosystems, particularly in a region recognized as a global biodiversity hotspot. By exploring the microbial communities in these unique and diverse environments, the study aims to fill a critical gap in current knowledge, shedding light on their ecological roles and potential implications for conservation. This work not only could enhance our understanding of freshwater microbiomes but also could emphasize the importance of safeguarding such biodiverse areas in the face of environmental changes.

## 2 Materials and Methods

### 2.1 Water Sampling and eDNA Extraction

**FIGURE 1:**
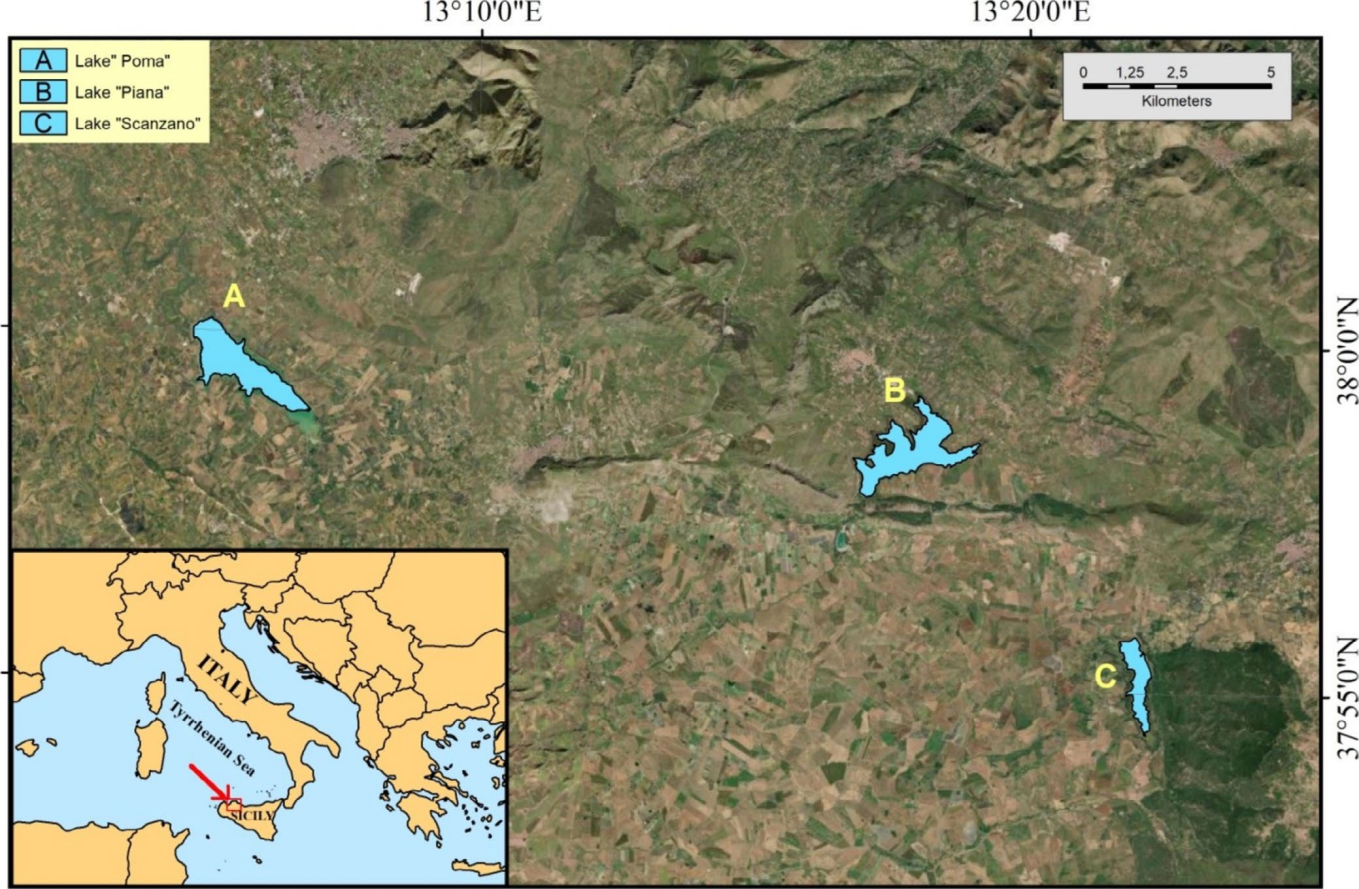
Location of the artificial basins analyzed in this study to collect eDNA: lake Poma, lake Piana, and lake Scanzano.

Environmental DNA (eDNA) was extracted from samples collected in October and November 2022 from three artificial basins in the province of Palermo: Poma lake, Scanzano lake, and Piana Albanesi lake (from now named Piana) (Figure 1). Lake Poma is in the Jato River basin within the territories of Monreale and Partinico and was formed by damming the Jato River. The lake's water is used for both irrigation and potable purposes, has a perimeter of about 12 km, and a surface area of 268 ha. Lake Piana is located in the Belice River basin, within the municipality of Piana degli Albanesi in Palermo. This lake was created by damming the Belice Destro River and is primarily used for energy production, irrigation, and water supply to Palermo. Lake Piana has a perimeter of 7.6 km and a surface area of 101 ha. Lake Scanzano, located in the Eleuterio River basin and spanning the municipalities of Monreale and Piana degli Albanesi in Palermo, was created by damming the Rossella Scanzano stream. It has a perimeter of 16.6 km and a surface area of 289 ha. Lake Piana and lake Scanzano are part of agricultural and urban contexts, and the water is used for irrigation of the fields. Their water supply derives from rainwater and river inflow. Water samples (2 L each) were collected in triplicate from the central point where all currents converged, near the dams, at the middle of the water column using sterile glass bottles (autoclaved and cleaned with 10% HCl). The water samples were kept cool and dark in refrigerated containers until arriving in the laboratories of the STEBICEF Department. Then, they were filtered in a sterile environment using nitrocellulose membranes (MF‐Millipore, 0.22 μm MCE Membrane, 47 mm, Merck, GSWP04700). In particular, one filter was used for each replicate, and MilliQ water was filtered to monitor contamination during the filtration process. The filters were cut into small strips, and eDNA was extracted using DNeasy Blood & Tissue Kits (Qiagen) and stored at −20°C. In each lake, pH, conductivity, and temperature were measured (Table 1).

**TABLE 1:** pH, conductivity, and temperature of each lake.

	Poma	Piana	Scanzano
pH	7.81	7.7	7.7
Conductivity (ppm)	190	173	179
Temperature (°C)	24	21.2	22.6

### 2.2 eDNA Analysis and Statistical Analysis

Metabarcoding analysis was performed by IGA Technology Services s.r.l. A two‐step PCR amplification was carried out. In the first step, specific primers to amplify the V3‐V4 region of the 16S rRNA gene were used. Illumina adapter overhang nucleotide sequences were added to the gene‐specific sequences. The full‐length primer sequences are the following: 16S‐515F 5′‐TCGTCGGCAGCGTCAGATGTGTATAAGAGACAGGTGYCAGCMGCCGCGGTAA‐3' 16S‐805R 5'‐GTCTCGTGGGCTCGGAGATGTGTATAAGAGACAGGGACTACNVGGGTWTCTAAT−3'. The PCR mix (final volume of 25 μL) contained 12.5 μL 2× KAPA HiFi HotStart ReadyMix and 2.5 μL of each 2 μM forward and reverse primer. After adding 50 ng of the DNA extract, this mix was incubated with initial denaturation for 3 min at 95°C, 25 cycles of denaturation for 30 s at 95°C, annealing for 30 s at 55°C, and extension for 30s at 72°C, and a final extension for 5 min at 72°C. PCR products were purified using 0.8X Ampure XP beads and eluted in 35 μL Tris–HCl pH 8.0 buffer. For the second step, the index PCR, 7.5 μL of the purified PCR product was added to a PCR mix containing 12.5 μL 2× KAPA HiFi HotStart ReadyMix and 2.5 μL of each index primer (Nextera XT). PCR conditions were: initial denaturation for 3 min at 95°C, 9 cycles of denaturation for 30 s at 95°C, annealing for 30 s at 55°C, and extension for 30 s at 72°C, and a final extension for 5 min at 72°C. After measuring with the Qubit 1X dsDNA HS Assay Kit (Thermo Fisher Scientific, Waltham, MA), the indexed PCR products were equimolarly pooled and sent for sequencing using the Illumina MiSeq 2x300 bp platform (Illumina, San Diego, CA). Base calling, demultiplexing, and adapter masking were performed on‐instrument with MiSeq Reporter.

Bioinformatics analysis was performed using the DADA2 pipeline for amplicon sequence variant (ASV) identification and taxonomic classification (Callahan et al. 2016). As the first step of the analysis, only pairs of reads containing both primers were selected using cutadapt v4.1 with parameters ‐‐overlap 10, ‐‐times 2, ‐‐error‐rate 0.15, ‐‐trim‐n, and ‐‐max‐n 0. ASV analysis was conducted using the DADA2 v1.22.0 and phyloseq v1.38.0 packages in R v4.1.3. Taxonomic classification up to the genus level was achieved using the Silva v138 database and the Bayesian classifier of the Ribosomal Database Project (RDP) available in the DADA2 package (kmer size 8, bootstrapping iteration: 100, bootstrapping confidence: 50). Species‐level classification was determined by exact matches between ASVs and a database of sequences defining known genus‐species associations, based on the Silva v138 database, accessible at this link. Raw reads were obtained for each sample, with an average of 719,040 reads (ranging from 145,386 to 1,125,232). After filtering, trimming, and merging steps, the number of featured reads retained for taxonomic annotation averaged 138,235 (ranging from 17,490 to 21,332; Supporting Information S1). The sequence dataset was deposited in the database GenBank (BioProject n. PRJNA1156621). To avoid a bias effect related to the excessive differences (dominance) in the number of identified fragments between the different taxa (the main bacterial phyla and families) and between the different sampling sites, the collected data were log‐transformed and then the relative abundance per site was calculated. Using only the qualitative‐quantitative transformed data of wild taxa in the different sampling sites the biodiversity indices (H′) with the Shannon algorithm were computed. The community found in the different sampling sites were compared by the similarity with qualitative‐quantitative values with the Bray‐Curtis index.

## 3 Results and Discussion

### 3.1 Bacterial Taxonomic Composition in the Three Sicilian Lakes

**FIGURE 2:**
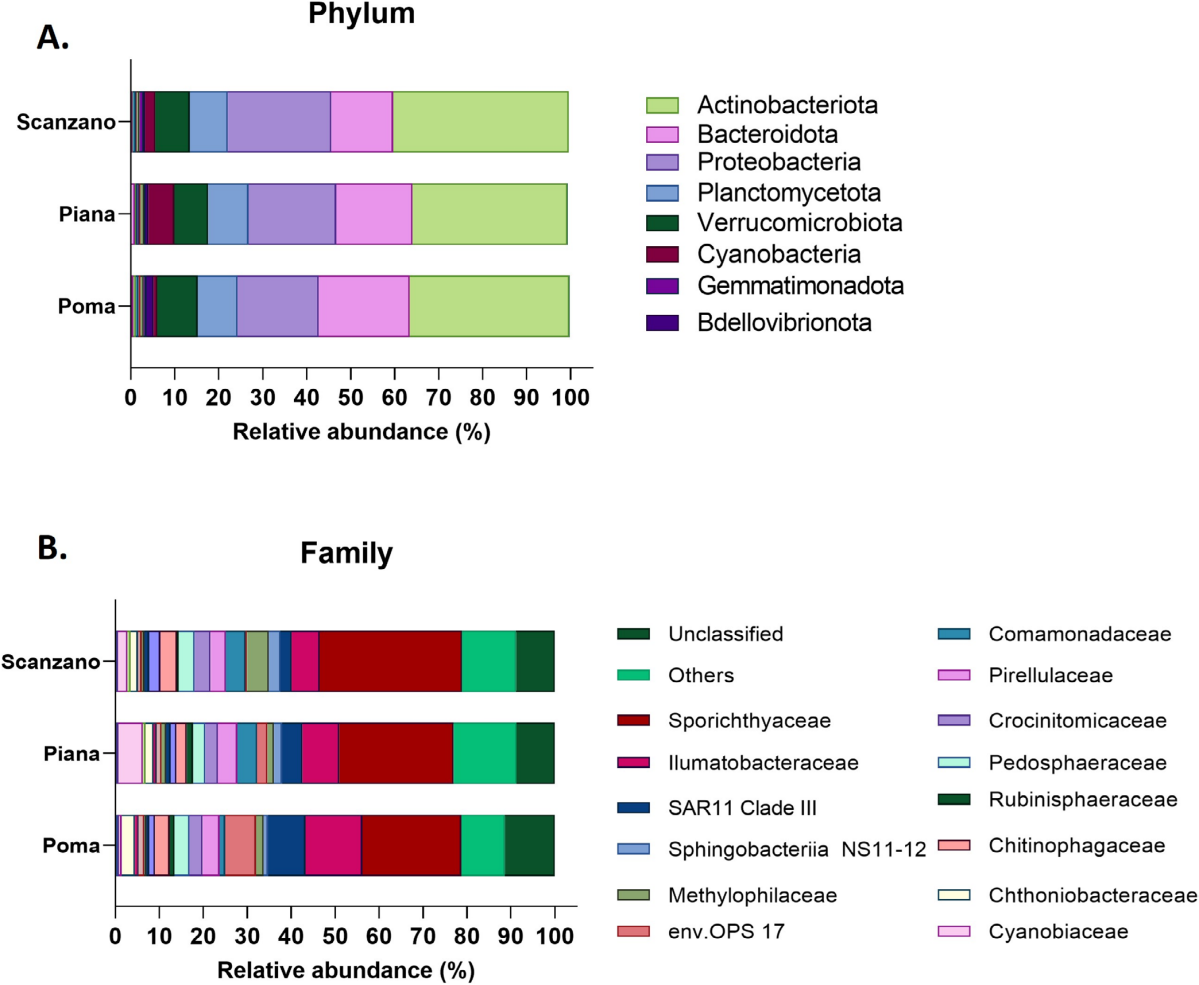
Relative abundance of bacteria in the three lakes (Poma, Piana, and Scanzano) at phylum (A) and family level (B). “Others” indicate the bacterial taxa with a relative abundance of < 0.5%.

In our study, eDNA‐based microbiota analysis provided valuable insights into the diversity and composition of microbial communities in Sicilian lakes. This approach identified 21 bacterial phyla, with five core phyla—Actinobacteriota, Proteobacteria, Bacteroidota, Planctomycetota, and Verrucomicrobiota—being notably predominant. The remaining 16 detected phyla included Cyanobacteria, Gemmatimonadota, Bdellovibrionota, Chloroflexi, Patescibacteria, Armatimonadota, Acidobacteriota, SAR324 clade, Desulfobacterota, Myxococcota, Dependentiae, Firmicutes, Spirochaetota, Nitrospinota, Crenarchaeota, and Nitrospirota (Figure 2A).

These findings highlight the complexity and richness of microbial communities thriving within these aquatic ecosystems. In addition, these results underscore the specificity of microbial components in environmental water samples, with each water characterized by its signature; as an example, Proteobacteria are always dominant in seawater samples (Sucato et al. 2021), while Proteobacteria and Bacteroidota in wastewaters (Di Leto et al. 2022) and this study, for the first time, demonstrate that Actinobacteriota can be considered the bacterial signature of lake samples. Actinobacteriota are usually abundant in soil (Catania et al. 2022); thus, this result strongly suggests their presence could be due to the leaching of water from the surrounding lands, especially in the case of tourist‐impacted lakes, such as lakes Poma, Piana, and Scanzano. However, given their ability to produce secondary metabolites, such as antibacterials (De Simeis and Serra 2021), Actinobacteriota could drive the bacterial colonization of the lake water, inhibiting the growth of some specific bacterial species. Small differences were also observed between the three lakes, with varying relative abundances of Actinobacteriota, Proteobacteria, and Bacteroidota. Actinobacteriota had a higher concentration in lake Scanzano (39.96% ± 8.25%) followed by lake Poma (36.26% ± 4.85%) and Piana (35.19% ± 4.13%). Similarly, Proteobacteria were more abundant in lake Scanzano (23.57% ± 0.5%) followed by lakes Piana (19.93% ± 0.18%) and Poma (18.5% ± 0.23%) which showed a similar relative abundance. Finally, Bacteroidota were more abundant in lake Poma (20.67% ± 5%) compared to lake Piana (17.35% ± 1.32%) and lake Scanzano (14.02% ± 1.45%).

The relative abundance of bacterial families of three samples was also evaluated (Figure 2B). Specifically, the three lakes were dominated by Sporichthyaceae and Ilumatobacteraceae (both belonging to the Actinobacteriota), and SAR11 Clade III (Proteobacteria). In particular, the Sporichthyaceae family showed a relative abundance of 32.36% ± 4.91% in lake Scanzano, 26.03% ± 4.3% in lake Piana, and 22.49% ± 3.02% in lake Poma. The Ilumatobacteraceae family was detected at 12.97% ± 1.75% in lake Poma, 8.43% ± 0.74% in lake Piana, and 6.34% ± 3.19% in lake Scanzano. The SAR11 Clade III family was found at 8.54% ± 1.01% in lake Poma, 4.61% ± 0.55% in lake Piana, and 2.57% ± 1.88% in lake Scanzano. Members of Ilumatobacteraceae and Sporichthyaceae are often associated with aquatic environments, including freshwater systems (Wu et al. 2023). SAR11 Clade III members have also been detected in freshwater environments, such as lakes, rivers, and reservoirs (Newton et al. 2011). However, they are typically present in lower abundance compared to marine habitats.

Other families included env.OPS 17 (Bacteriota), Pirellulaceae (Planctomycetota), Pedosphaeraceae (Verrucomicrobiota), and Chitinophagaceae (Bacteriota). Env.OPS 17 family was present at 7.01% ± 2.25% in lake Poma, 2.39% ± 0.29% in lake Piana, and 0.37% ± 0.03% in lake Scanzano. Pirellulaceae family showed a relative more abundance in lake Piana (4.45% ± 1.14%) than in lakes Poma and Scanzano (3.84% ± 0.12% and 3.43% ± 2.2%, respectively). Pedosphaeraceae family was found at a similar relative abundance in lakes Poma and Scanzano (3.38% ± 0.28% and 3.65% ± 1.21%, respectively) in respect to lake Piana (2.87% ± 0.25%). Similarly, Chitinophagaceae family was detected in lakes Poma and Scanzano (3.36% ± 0.41% and 3.83% ± 0.23%, respectively) in comparison to lake Piana (2.3% ± 0.36%). The bacterial family env.OPS 17 has limited documentation in scientific literature and well‐characterized representatives are not known. Currently, only five cultured organisms belonging to this family have been described, associated with fungi or in freshwater springs (Wu et al. 2020). Pirellulaceae members are predominantly found in marine and freshwater environments, which are often associated with macroalgae (Brümmer et al. 2004; Bondoso et al. 2017; Wiegand et al. 2021; Tadonléké 2007).

### 3.2 Bray‐Curtis UPGMA and Heatmap Analysis

**FIGURE 3:**
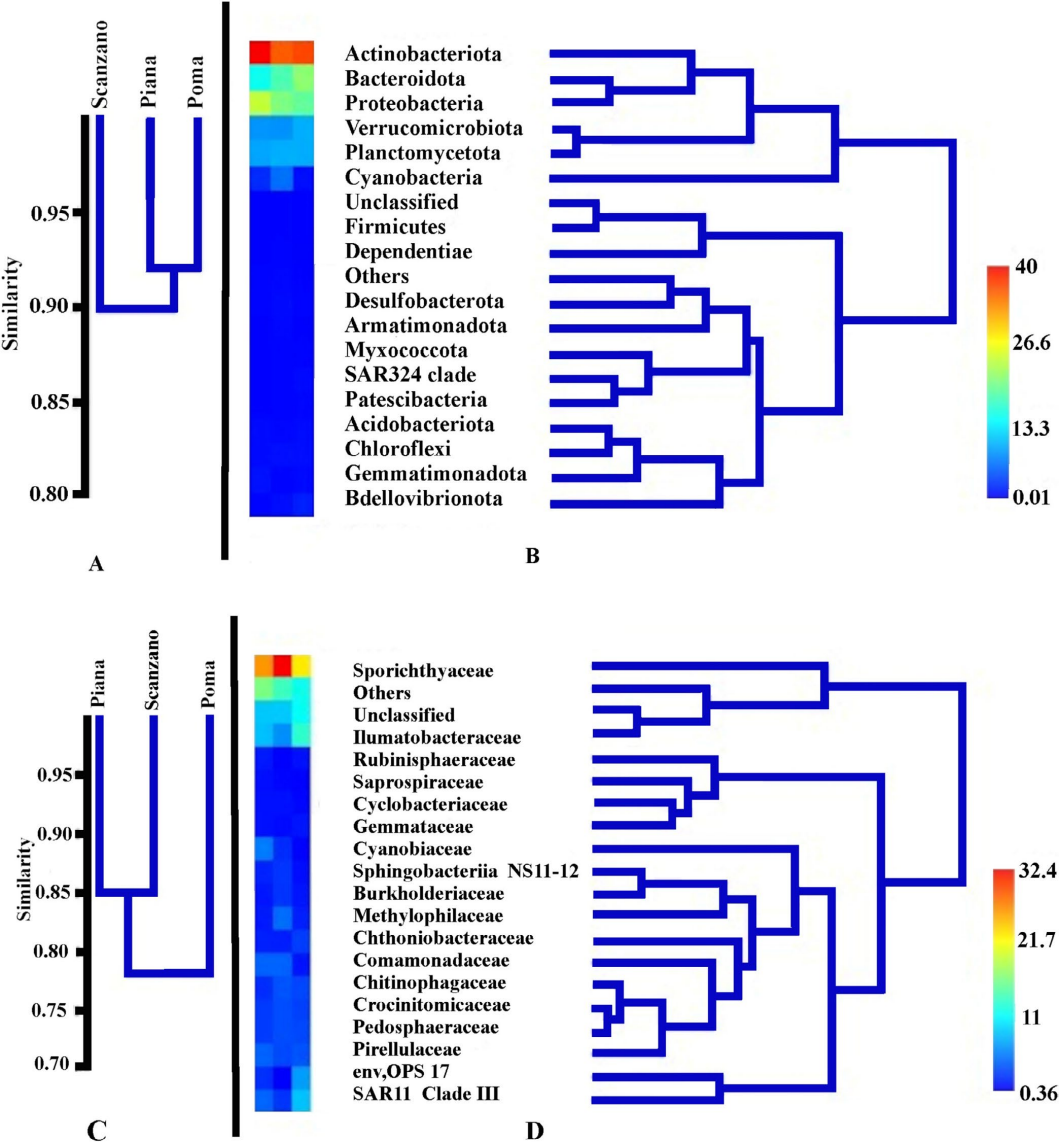
Dendrogram of Bray‐Curtis quali‐quantitative similarity among the three Sicilian lakes, constructed using the UPGMA method: (A) similarity at the phylum level; (B) dendrogram and heatmap depicting theBray‐Curtis similarity of the microbiota community at the phylum level; (C) similarity at the family level; (D) dendrogram and heatmap illustrating theBray‐Curtis similarity of the microbiota community at the family level.

The dendrogram generated using the Bray‐Curtis index with the UPGMA method (Figure 3A) and the heatmap visualization (Figure 3B) revealed greater microbial diversity in lake Scanzano compared to the other lakes. The heatmap on the left displays the relative abundance of bacterial phyla across different sampling sites, with a color gradient representing abundance levels (warmer colors indicate higher abundance, cooler colors reflect lower abundance). lakes Poma and Piana showed more similarity, as evidenced by their shorter distance on the dendrogram relative to lake Scanzano. This proximity suggests a greater degree of similarity in their microbial community compositions (Figure 3A).

By combining the dendrogram and heatmap, we gained a more detailed and nuanced understanding of the phylum discrimination patterns among the samples, which enhanced the overall interpretation of microbial diversity and community composition. These patterns offer valuable insights into the unique structure and composition of the microbial communities in each sample. Specifically, samples from lakes Poma and Piana exhibited more similar phylum profiles, which led to their clustering together in the analysis (Figure 3A,B).

Actinobacteriota and Proteobacteria showed the highest relative abundance in lake Scanzano, whereas Bacteroidota was more prevalent in lakes Piana and Poma. Verrucomicrobiota reached its highest abundance in lake Poma, exceeding levels observed in lakes Scanzano and Piana. Similarly, Cyanobacteria were more abundant in lake Piana than in lakes Poma and Scanzano. Planctomycetota, Firmicutes, and Acidobacteria displayed slight variations across the lakes (Figure 3B).

Figure 3C,D, represent the dendrogram obtained with the Bray‐Curtis index UPGMA method at the family level. Here, while the similarity values remain high, they are somewhat lower compared to the phylum‐level comparison, likely due to the finer resolution provided by higher taxonomic classification. In particular, the dendrogram analysis at the family level showed lakes Piana and Scanzano with a more similar clustering pattern to each other than to lake Poma (Figure 3C,D). Figure 3D displays a heatmap and a dendrogram illustrating the bacterial family composition and diversity in the three Sicilian lakes (Poma, Piana, and Scanzano). The dendrogram on the right side illustrates the hierarchical clustering of the sampling sites based on their bacterial family composition. Lakes Piana and Scanzano are closely clustered together, suggesting a high degree of similarity in their family composition. This similarity might be attributed to comparable environmental conditions or similar anthropogenic influences impacting these two lakes. Lake Poma, while showing some similarity to lakes Scanzano and Piana, forms a slightly distinct cluster, indicating differences in its microbial community structure. These differences could be due to unique local factors, such as variations in water chemistry, nutrient availability, or specific anthropogenic activities.

Notable families, such as Sporichthyaceae, Ilumatobacteraceae, SAR11 clade III, env.OPS 17, and Cyanobiaceae showed significant variation in their presence across the sites. For instance, the Sporichthyaceae family was more abundant in lake Scanzano, followed by lakes Poma and Piana. Ilumatobacteraceae and SAR11 clade III families were more abundant in lake Piana than in lakes Scanzano and Poma. The Cyanobiaceae family was more abundant in lake Piana. Finally, env.OPS 17 was more abundant in lake Scanzano.

### 3.3 Physicochemical Parameters in Relation to Bacterial Biodiversity

The physicochemical properties of a lake play a crucial role in shaping its biodiversity, including microbial diversity and overall species richness. In particular, pH, conductivity, and temperature are considered key drivers of microbial community variations (Pu et al. 2023; Guo et al. 2024; Bååth and Kritzberg 2024). The recorded pH, conductivity, and temperature in the three lakes Poma, Piana, and Scanzano are shown in Table.

The pH values (7.7–7.81) indicate that all three lakes are slightly alkaline. This pH range is generally favorable for aquatic biodiversity, as it supports the growth and survival of most freshwater species, including plankton, invertebrates, and fish, which typically thrive in waters with a pH between 6.5 and 8.5.

Conductivity values (173–190 ppm) indicate the ionic concentration of the water, which correlates with nutrient levels and potential organic matter input. The values in this study are relatively low, suggesting that the lakes are not experiencing excessive nutrient pollution or salinity issues (Muscarella et al. 2024; Cusenza et al. 2025). Lake Poma (190 ppm) has the highest conductivity, which could indicate slightly higher dissolved ion concentrations, potentially linked to greater biological activity or minor anthropogenic influences (e.g., agricultural runoff or wastewater input). In general, these values suggest moderate trophic conditions in the three lakes. The recorded temperatures (21.2°C–24°C) suggest warm, mesotrophic conditions, typical of Mediterranean lakes (Giadrossich et al. 2017). Lake Poma (24°C) has the highest temperature, followed by lakes Scanzano (22.6°C) and Piana (21.2°C).

**FIGURE 4:**
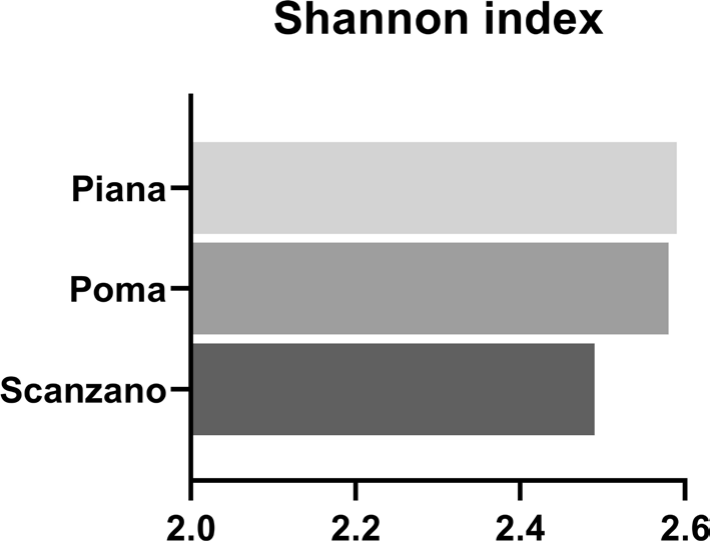
Comparison of Shannon index at the family level in three lakes.

Shannon index was also calculated to determine the family diversity in the different samples. The three lakes showed a similar family diversity (i.e., Shannon index of about 2.5–2.6) with a slightly lower value in lake Scanzano (Figure 4). The Shannon Index of 2.5–2.6 aligns with biodiversity levels found in mesotrophic freshwater lakes and indicates a moderate‐to‐high bacterial diversity (Wang et al. 2022).

## 4 Conclusions

This study provides an extensive examination of microbial diversity in three Sicilian lakes: Poma, Piana, and Scanzano. Through the use of eDNA analysis and high‐throughput next‐generation sequencing, we have successfully mapped the microbial communities within these freshwater systems. Our findings show that while the three lakes share a broadly similar bacterial composition, lakes Poma and Piana are more similar to each other than to lake Scanzano. However, environmental factors, such as temperature, light intensity, and pH could have influenced eDNA degradation, impacting the accuracy and detectability of the microbial profiles. The interaction of these factors may cause eDNA to degrade rapidly, with some studies indicating a degradation window as short as 1–2 days (Zulkefli et al. 2019).

Despite these limitations, our study highlights the tremendous potential of eDNA as a rapid, non‐invasive tool for biodiversity assessment and environmental monitoring. The findings offer valuable insights into the microbial dynamics of freshwater ecosystems, underscoring the importance of incorporating microbial diversity into conservation and management strategies. Looking ahead, this research could pave the way for broader applications of eDNA in biodiversity monitoring, providing a powerful tool for tracking changes in ecosystem health, especially in response to environmental pressures, such as climate change and pollution. Additionally, further studies exploring the interaction between eDNA degradation and environmental variables could help refine and optimize eDNA‐based methods, enabling more accurate and reliable biodiversity assessments. Integrating microbial community data with traditional water quality indicators could lead to more holistic and informed management practices, ensuring the long‐term resilience and sustainability of freshwater ecosystems.

## Author Contributions

**Manuela Mauro:** investigation (lead), methodology (lead), writing – original draft (lead). **Valeria Villanova:** investigation (lead), validation (lead), writing – original draft (lead). **Mario Lo Valvo:** methodology (lead), validation (lead), writing – review and editing (lead). **Rosa Alduina:** data curation (lead), validation (lead), writing – review and editing (lead). **Slobodanka Radovic:** investigation (equal). **Aiti Vizzini:** methodology (equal). **Grazia Orecchio:** methodology (equal). **Francesco Longo:** methodology (equal). **Rosario Badalamenti:** methodology (equal). **Vincenzo Ferrantelli:** methodology (equal). **Gaetano Cammilleri:** methodology (equal). **Annamaria Mauro:** methodology (equal). **Vincenzo Arizza:** funding acquisition (lead), resources (lead), writing – review and editing (equal). **Mirella Vazzana:** funding acquisition (equal), resources (equal), supervision (lead), validation (lead), writing – review and editing (equal).

## Conflicts of Interest

The authors declare no conflicts of interest.

## Supporting information


Appendix S1:

## Data Availability

The sequence dataset was deposited in the database GenBank (BioProject n. PRJNA1156621).
